# High-intensity interval training reduces Tau and beta-amyloid accumulation by improving lactate-dependent mitophagy in rats with type 2 diabetes 

**DOI:** 10.22038/ijbms.2024.77038.16664

**Published:** 2024

**Authors:** Pouria Khosravi, Fereshte Shahidi, Arezoo Eskandari, Kayvan Khoramipour

**Affiliations:** 1 Department of Sports Physiology, Faculty of Physical Education and Sports Sciences, Shahid Rajaee Teacher Training University, Tehran, Iran; 2 i+HeALTH Strategic Research Group, Department of Health Sciences, Miguel de Cervantes European University (UEMC), 47012 Valladolid, Spain

**Keywords:** Cognitive dysfunction, Diabetes mellitus-type 2, High-intensity interval – training, Hippocampus, Lactates, Mitochondria, Mitophagy

## Abstract

**Objective(s):**

This study aimed to investigate the effect of 8-week high-intensity interval training (HIIT) on lactate-induced mitophagy in the hippocampus of rats with type 2 diabetes.

**Materials and Methods:**

28 Wistar male rats were divided into four groups randomly: (i) control (Co), (ii) exercise (EX), (iii) type 2 diabetes (T2D), and (iv) type 2 diabetes + exercise (T2D + Ex). The rats in the T2D and T2D + Ex groups were fed a high-fat diet for two months, then a single dose of STZ (35 mg/kg) was injected to induce diabetes. The EX and T2D + Ex groups performed 4–10 intervals of treadmill running at 80–100% of Vmax. Serum and hippocampal levels of lactate, as well as hippocampal levels of monocarboxylate transporter2 (MCT2), sirtuin1 (SIRT1), forkhead box protein O (FOXO3), light chain 3 (LC3), PTEN-induced kinase 1 (PINK1), parkin, beta-amyloid (Aβ), hyperphosphorylated tau protein (TAU), Malondialdehyde (MDA), and antioxidant enzymes were measured. One-way ANOVA and Tukey post-hoc tests were used to analyze the data.

**Results:**

Serum and hippocampal levels of lactate as well as hippocampal levels of MCT2, SIRT1, FOXO3, LC3, PINK1, Parkin, and antioxidant enzymes were higher while hippocampal levels of Aβ, TAU, and MDA were lower in T2D+EX compared to T2D group (*P*-value<0.05)

**Conclusion:**

HIIT could improve mitophagy through Lactate-SIRT1-FOXO3-PINK1/Parkin signaling in the hippocampus of rats with T2D reducing the accumulation of Tau and Aβ, which may reduce the risk of memory impairments.

## Introduction

Graphical abstract. High-Intensity Interval Training (HIIT), SIRT1 (Sirtuin1), Forkhead box protein O (FOXO3), Amyloid-β (Aβ), Tau Protein (Tau), PTEN-induced kinase 1 (PINK1), Monocarboxylate transporter 2 (MCT2), Superoxide dismutase (SOD), Glutathione Peroxidase (GPx), Malondialdehyde (MDA)

Mitochondria are essential organelles responsible for a myriad of cellular processes including replication and transcription of their own DNA, translation of mitochondrial proteins, turnover of oxidants, importation of nuclear-encoded proteins, storage of calcium ions, and regulation of apoptosis (1-3). Maintaining a robust population of mitochondria is crucial for cellular functionality and longevity. This equilibrium is achieved through the delicate regulation of mitochondrial biogenesis and turnover, primarily mediated by autophagy, specifically referred to as mitophagy (4, 5). Mitophagy represents a selective mechanism wherein damaged mitochondria are degraded via autophagy, facilitating the removal of dysfunctional mitochondria from the cellular milieu. This process is notably triggered in response to various stressors such as starvation, ischemia/reperfusion injury, and pathogenic infections. Pertinently, disruptions in mitophagy have been implicated in metabolic disorders like diabetes, potentially contributing to associated complications. Notably, impaired mitophagy within the hippocampus has been linked to the accumulation of beta-amyloid and tau proteins, pivotal markers associated with cognitive decline and impairment (6). Impairment in mitophagy exacerbates the accumulation of dysfunctional mitochondria within the cell, thereby amplifying the buildup of reactive oxygen species (ROS) and diminishing the mitochondrial membrane potential. This cumulative effect significantly contributes to mitochondrial damage, compromising cellular health and function (7, 8). Beta-amyloid (Aβ) and tau proteins can adhere to certain membrane surface proteins of damaged mitochondria. This interaction induces oxidative stress and interferes with calcium uptake, disrupting the electron transport chain and causing alterations in mitochondrial DNA (mtDNA). Consequently, these cascading effects ultimately culminate in cellular dysfunction and eventual cell death (9, 10). 

Recent studies have provided evidence indicating that lactate has the capacity to stimulate mitophagy (11, 12). However, despite the observed elevation in lactate levels in the serum of diabetic individuals, mitophagy impairment has been reported in this population. This discrepancy may be elucidated by a deficiency in monocarboxylate transporters (MCTs) observed in diabetes, which could hinder the effective transport of lactate and subsequently impact mitophagy processes (13). Shima *et al*. conducted a study demonstrating that exercise activity has the potential to ameliorate cognitive impairments in mice afflicted with type 2 diabetes. This effect was attributed to the up-regulation of monocarboxylate transporter 2 (MCT2) expression in the hippocampus (14).

Lactate can cross the blood-brain barrier (BBB) via MCT2 and penetrate various brain regions, including the hippocampus. Upon entering the hippocampus, lactate can elevate sirtuin 1 (SIRT1) levels. It has been elucidated that lactate serves as one of the factors capable of enhancing SIRT1 expression (15). Previous studies showed that exercise significantly alters the NAD+/NADH ratio, which leads to increased expression of SIRT1 in the brain (12, 16). During mitophagy, the cytosolic form of LC3 (microtubule-associated protein 1A/1B-light chain 3) conjugates with phosphatidylethanolamine to form LC3-II, which is then recruited to autophagosome membranes. The increase in LC3-II levels indirectly indicates the activation of mitophagy, serving as a marker for autophagosome formation and maturation (17).

SIRT1 has the capability to enhance the expression of Forkhead box protein O (FOXO3), a pivotal transcriptional regulator involved in orchestrating cellular responses to various stimuli. FOXO3 serves as a central hub for integrating multiple cellular signals and is known to stimulate the expression of antioxidants, thereby contributing to cellular defense mechanisms against oxidative stress (18). On the contrary, elevating SIRT1 levels can mitigate the production of ROS (19). Past studies have indicated that NAD+-dependent SIRT1 enhances mitophagy by activating the FOXO1/3-PINK1-Parkin pathway (20, 21). Parkin plays a pivotal role as a mediator of mitophagy, and its function is intricately intertwined with that of PINK1 (22). FOXO3 has the ability to activate the PINK1/Parkin pathway, thereby augmenting mitophagy (20). Research findings have established a close relationship between oxidative stress and mitophagy. In a study assessing markers of oxidative stress, such as malondialdehyde (MDA), and their correlation with mitophagy in hippocampal tissue, it was noted that elevated MDA levels were associated with heightened mitochondrial damage and diminished mitophagy activity (23). In diabetic rats, there is a notable elevation in oxidative stress levels. This increase in oxidative stress can precipitate the degradation of healthy mitochondria (24). The accumulation of dysfunctional mitochondria can indeed lead to the accumulation of tau and Aβ proteins (24).

Indeed, exercise has been demonstrated to enhance mitophagy by improving oxidant defense mechanisms and reducing the production of ROS (12, 25-27). However, the specific role of lactate as a mediator in exercise-induced mitophagy has not yet been thoroughly investigated.

In this study, we investigated the impact of high-intensity interval training (HIIT)-induced lactate accumulation on enhancing mitophagy via the SIRT1-FOXO3-PINK1-Parkin cascade in the hippocampus of type 2 diabetic rats. Our hypothesis posited that enhancing mitophagy could mitigate the accumulation of Aβ and tau proteins in the hippocampus of these animals. 

## Materials and Methods

Twenty eight male Wister rats were bought from the animal farm of Kerman University of Medical Sciences. The rats had an average weight of 200±10.25 g and were 8 weeks old. The rats were housed in polycarbonate cages that were maintained at a controlled temperature of 22 ± 1.4 °C, a humidity level of 50 ± 4, and a light cycle of 12 hr of light followed by 12 hr of darkness. The rats were given free access to food and water. 

***Animal grouping***

Initially, the animals were acclimatized to the laboratory for one week and allocated into four groups, each consisting of 7 rats:(i) CO (healthy control), (ii) EX (exercise), (iii) T2D (type 2 diabetes), and (iv) T2D+EX (type 2 diabetes + exercise).

***Type 2 diabetes*** ***Induction***

During the initial eight-week period, the animals belonging to the T2D and T2D+EX groups were provided with a high-fat diet (HFD) (60% fat, 20%protein, and 20% carbohydrate) (28, 29). Following the eight-week period, the rats were fasted for 12 hr before being injected intraperitoneally with a single dose of 35 mg/kg streptozotocin (STZ). Three days after the injection, the rats’ blood glucose levels were measured using a glucometer (30, 31). The inclusion criterion for the rats to be a part of the study was that their fasting blood glucose (FBG) level must be above 300 mg/dl, which indicated that they had diabetes (32-34).

***Exercise protocol***

All rats underwent a five-day familiarization period on a treadmill, during which they ran at a speed of 8 m/min for 10 min. Following this familiarization period, an incremental test was conducted to determine the rats’ maximum velocity (Vmax). During this test, the rats ran for 2 min at a speed of 6 m/min, and every 2 min, the treadmill speed was increased by 2 m/min until the rats were exhausted. The last speed that the rats could tolerate was considered their Vmax. The training protocol is modified every two weeks based on the rats’ updated Vmax measurements (35-46). 

***Sampling***

*Blood sample*

After 48 hr of the last training session, the rats were euthanized using anesthesia, which was administered through intraperitoneal injection of a combination of ketamine (80 mg/kg) and xylazine (10 mg/kg). Blood samples were obtained from the rats’ hearts and were centrifuged at 1000 g for 20 min at a temperature of 4 °C, and the resulting serum samples were stored at a temperature of -80 °C until they could be analyzed (35, 36). 

*Hippocampus sample*

Additionally, the hippocampus of the rats was extracted and washed in a solution of PBS. The tissue was then homogenized using an ultrasonic homogenizer in Ripa buffer solution with protease inhibitor on ice. The resulting homogenate was centrifuged at 13,000 rpm for 20 min at a temperature of 4 °C, and the supernatant was collected and stored at a temperature of -80 °C until it could be analyzed.

***Western blot***

To determine the concentrations of Monocarboxylate Transporter2 (MCT2) (sc-166925, SANTA CRUZ BIOTECHNOLOGY, INC.), Sirtuin1 (SIRT1)( EPR18239,abcam), Forkhead box protein O (FOXO3) (sc-48348m, SANTA CRUZ BIOTECHNOLOGY, INC.), Light Chain 3 (LC3)(#2775, cell signaling technology), PTEN-induced kinase 1 (PINK1) (sc-517353, SANTA CRUZ BIOTECHNOLOGY, INC.), Parkin (sc-32282, SANTA CRUZ BIOTECHNOLOGY, INC.), Amyloid Beta (Aβ) (sc-28365, SANTA CRUZ BIOTECHNOLOGY, INC.), and hyperphosphorylated tau protein (TAU) (sc-21796, SANTA CRUZ BIOTECHNOLOGY, INC.) the western blotting method was employed. To perform this test, the samples were prepared and the target protein was separated on a gel, which was then transferred to a nitrocellulose paper with a pore size of 0.45 μm at a current of 0.5 amps for 1.5 hr. The gel was placed in a buffer for at least 10 min after the compacting part was cut. Precisely sized nitrocellulose paper and filter pads were cut using pliers and placed on either side of the membrane and gel. The blot sandwich was then placed in a plastic frame and immersed in a buffer-filled blot tank to the appropriate height. The primary antibodies, diluted at a ratio of 1:1000, were incubated for 16 to 18 hr. The membrane was then placed in a secondary antibody solution with an appropriate concentration in EIA buffer for 1.5 hr, after which it was shaken twice with TBST and once with PBS. The membrane was then placed in an acceptable TMB substrate solution until bands appeared, and the reaction was stopped by adding distilled water. The Chemi Doc XRS + imaging system (Bio-Rad, USA) was used to record the results, which were analyzed using Image J software. Beta-actin protein was used as a control for the analysis (47, 48).

***ELISA***

The ELISA method was utilized to measure the concentration of lactate in the serum and hippocampal tissue and serum insulin levels in accordance with the standard commercial kit. The concentration of Lactate in the hippocampal tissue and serum was divided by the total protein concentration (mg/ml) of the respective homogenates. The hippocampus was washed using a saline solution and Trigger buffer (Sigma), after which it was homogenized for 5 min using a 5000 rpm homogenizer. The homogenized solution was then centrifuged using a refrigerated centrifuge. To prevent enzyme and protein degradation, all steps were performed at a temperature of 4 °C (49). A protease inhibitor solution of 0.5 mM phenylmethylsulfonyl fluoride was used. After centrifugation, the upper portion of the solution was removed using a sampler, and the amount of Lactate tissue was measured using the ELISA method and an ELISA reader device. The results were presented in nanograms per milligram of protein lactate using the Rat ELISA Kit (Eastbiopharm) (50, 51).

***MDA levels in the hippocampus***

MDA, which is the result of peroxidation of membrane lipids, is considered an oxidant that will be measured using the thiobarbituric acid method. The hippocampal tissue placed in physiological saline solution at cold temperature was homogenized with a homogenizer and subsequently centrifuged at 1000 revolutions per minute for 10 min to produce a supernatant liquid. Then 20 microliters of the sample were added to a reaction mixture containing 150 microliters of thiobarbituric acid, 20 microliters of sodium dodecyl sulfate (SDS), 150 microliters of 20% acetic acid (pH = 3.5), and 60 microliters of distilled water. The resulting mixture was heated at 90 degrees Celsius for 45 min. After cooling it to room temperature for 10 min, it was then centrifuged at 10000 g to obtain a clear solution. Absorption was then recorded at 532 nanometers. In this experiment, tetramethoxypropane was used as a standard in such a way that solutions with concentrations of 2.5, 5, 10, 20, 30, 40, and 50 nanomoles per milliliter of this standard were prepared and after performing the above steps, a standard curve was plotted and the amount of lipid peroxidation was expressed in nanomoles of MDA per milliliter using the standard curve (52).

***Measurement of SOD activity in the hippocampus***

The amount of superoxide dismutase activity was measured indirectly using a colorimetric method based on SOD’s ability to inhibit the auto-oxidation of pyrogallol. For this purpose, 50 milligrams of hippocampal tissue was homogenized in 250 microliters of lysis buffer. The homogenate was then centrifuged at 12000 revolutions per minute for 5 min at 4 degrees Celsius, and the supernatant was used to measure the amount of SOD activity according to the kit instructions.

***Measurement of GPx activity in the hippocampus***

Glutathione peroxidase activity was based on glutathione peroxidase’s ability to oxidize glutathione (GSH) to oxidized glutathione (GSSG). GSSG is part of the reactions that reduce common hydroperoxide. Glutathione reductase then converts GSSG back to GSH by consuming nicotinamide adenine dinucleotide phosphate (NADPH). The reduction of NADPH, which is measured at 340 nanometers, is an indicator of glutathione peroxidase activity.

***Calculation of insulin resistance/sensitivity indices***

The homeostasis model assessment (HOMA) was utilized to evaluate insulin resistance (HOMA-IR) and homeostasis model assessment of β-cell function (HOMA-β). The HOMA-IR and HOMA-β scores were obtained using the following formula: HOMA-IR = [(fasting glucose (mmol/l) × fasting insulin (μU/ml))/22.5). HOMA-β-cell = [(20 × fasting insulin (μU/ml))/(fasting glucose (mmol/l) – 3.5) (35). The quantitative insulin sensitivity check index (QUICKI) was also used since it has a better linear correlation with glucose clamp determinations of insulin sensitivity than minimal-model estimates. QUICKI was calculated using the formula proposed by Katz *et al*. (36), which is 1/[log (fasting insulin in μU/ml) + log (fasting glucose in mg/dl))(34). 

***Statistical analysis***

The normality and homogeneity of the data were assessed using the Shapiro-Wilk and Levin tests, respectively. To compare the variables, one-way ANOVA was employed, and the Tukey test was utilized to identify significant differences between the groups. The data are presented as the mean ± standard deviation (SD). A *P*-value less than 0.05 was considered statistically significant. All statistical analyses were conducted using Graph Pad Prism 9.

## Results

***Animal weight and blood glucose***

We evaluated FBG to validate our method for inducing diabetes. The results of a One-way Analysis of Variance (ANOVA) revealed significant differences in both FBG (F _3,24 _=95.2, *P*<0.001) and body weight (F _3,24 _=95.2, *P*<0.001) among the various experimental groups. The outcomes of our study demonstrated a significant elevation in FBG levels following the induction of diabetes (2 months of a high-fat diet and streptozotocin injection) at month 2, compared to baseline levels (month 0) in both the T2D and T2D + EX groups (*P*=0.000), with no significant disparity observed between these groups. Furthermore, at month 4, the T2D+EX group exhibited a lower FBG level in comparison to the T2D group (*P*<0.000). The weight of the animals exhibited a notable increase in both the T2D and T2D + EX groups following diabetes induction (2 months of high-fat diet and streptozotocin injection) (*P*<0.000). Moreover, by month 4, a reduction in weight was observed in both groups (T2D and T2D+EX) (*P*<0.000), with a more pronounced decrease noted in the T2D group (*P*<0.000) (Figure 2).

***Insulin, insulin resistance/sensitivity indexes***

The findings from the One-way Analysis of Variance (ANOVA) revealed notable distinctions in serum insulin levels (F _3,24 _=95.2, *P*<0.001) across the experimental groups. Notably, there was a significant difference observed in serum insulin levels between the Con and T2D groups (*P*<0.05). Furthermore, the T2D+Ex group exhibited significantly elevated serum insulin levels compared to the T2D group (*P*<0.05). To evaluate the potential improvement in insulin sensitivity induced by T2D and Ex, we assessed insulin resistance/sensitivity indices, including HOMA-IR, HOMAβ, and QUICKI. The results of the One-way Analysis of Variance (ANOVA) revealed significant differences in HOMA-IR (F _3,24 _=124.5, *P*<0.0001) among the experimental groups. Specifically, our findings indicated that T2D and Ex led to increases and decreases in HOMA-IR, respectively (*P*<0.05). Additionally, the T2D+Ex group exhibited significantly reduced HOMA-IR levels compared to the T2D group (*P*<0.05). Moreover, significant differences were observed in HOMAβ (F _3,24 _=89.8, *P*<0.001) and QUICKI (F _3,24 _=156.69, *P*<0.0001) among the experimental groups. HOMAβ decreased following T2D induction and increased following Ex (*P*<0.05). Furthermore, the T2D+Ex group demonstrated lower HOMAβ levels compared to the T2D group (*P*<0.05). Additionally, a significant decrease was observed following T2D induction, while it increased following Ex. Furthermore, the T2D+Ex group exhibited lower QUICKI levels compared to the T2D group (*P*<0.05) (Figure 3). In summary, these findings indicate that T2D induction resulted in increased insulin resistance IR, while Ex intervention led to a decrease in IR (Figure 3).

***Molecular changes***

*Lactate levels in serum and hippocampus and MCT2 levels in the hippocampus*

The results of the One-way Analysis of Variance (ANOVA) indicated significant differences in serum lactate levels (F _3,24 _=104.3, *P*<0.0001) among the experimental groups. Particularly, there was a notable disparity observed in serum lactate levels between the CO and T2D groups (*P*<0.0001). Additionally, the T2D+Ex group exhibited significantly higher serum lactate levels compared to the T2D group (*P*<0.0001) (Figure 4).

The One-way Analysis of Variance (ANOVA) results showed significant variations in hippocampal lactate levels among the groups (F _3,24 _=160.1, *P*<0.0001). Specifically, a significant difference in hippocampal lactate levels was noted between the CO and EX groups (*P*<0.0001). Moreover, the T2D+Ex group displayed elevated levels of hippocampal lactate compared to the T2D group (*P*<0.0001) (Figure 3).

The One-way Analysis of Variance (ANOVA) results demonstrated significant disparities in MCT2 levels within the hippocampus across the groups (F _3,24 _=3970, *P*<0.0001). Notably, MCT2 levels in serum exhibited significant distinctions between the Control (CO) group and the other groups (*P*<0.0001). Furthermore, the T2D+EX group exhibited elevated MCT2 levels in the hippocampus compared to the T2D group (*P*<0.0001) (Figure 4).

*SIRT-1, FOXO-3 – PINK1 – Parkin pathway and LC3 levels in hippocampus*

The One-way Analysis of Variance (ANOVA) findings uncovered noteworthy disparities in SIRT-1 levels within the hippocampus across the groups (F _3,24 _= 334.1, *P*<0.0001). Particularly, SIRT-1 levels in the hippocampus were markedly elevated in the CO group compared to the T2D group (*P*<0.0001), and the T2D+Ex group exhibited significantly higher SIRT-1 levels in the hippocampus relative to the T2D group (*P*<0.0001) (Figure 5).

Similarly, the results of the One-way Analysis of Variance (ANOVA) revealed notable variations in FOXO-3 levels within the hippocampus among the groups (F _3,24 _=367.6, *P*<0.0001). Specifically, FOXO-3 levels in the hippocampus were significantly elevated in the CO group compared to the T2D group (*P*<0.0001), and the T2D+Ex group exhibited significantly higher FOXO-3 levels in the hippocampus compared to the T2D group (*P*<0.0001) (Figure 5).

Moreover, the One-way ANOVA findings underscored notable disparities in LC3 expression levels within the hippocampus among the experimental groups (F _3,24 _=3341, *P*<0.0001). Particularly striking was the substantially heightened expression of LC3 observed in the CO group in comparison to the T2D group (*P*<0.0001). Additionally, the T2D+Ex group exhibited a remarkable elevation in LC3 expression levels within the hippocampus compared to the T2D group (*P*<0.0001) (Figure 5).

Additionally, the One-way ANOVA findings revealed significant variations in PINK1 expression levels within the hippocampus among the experimental groups (F _3,24 _=85.70, *P*<0.0001). Notably, there was a substantial elevation in PINK1 expression levels observed in the CO group compared to the T2D group (*P*<0.0001). Furthermore, the T2D+Ex group exhibited significantly higher PINK1 levels in the hippocampus compared to the T2D group (*P*<0.0001) (Figure 5).

Conclusively, the One-way ANOVA findings unveiled significant discrepancies in Parkin expression levels within the hippocampus among the experimental groups (F _3,24 _=3873, *P*<0.0001). Notably, Parkin levels in the hippocampus were markedly elevated in the CO group compared to the T2D group (*P*<0.0001). Furthermore, the T2D+Ex group exhibited significantly higher Parkin levels in the hippocampus compared to the T2D group (*P*<0.0001) (Figure 5).

*Aβ and Tau levels in the hippocampus*

The One-way ANOVA results indicated significant differences in Aβ levels within the hippocampus among the groups (F _3,24 _=147, *P*<0.0001). Specifically, Aβ levels in the hippocampus differed significantly between the CO and T2D groups (*P*<0.0001). Moreover, the T2D+Ex group exhibited lower Aβ levels in the hippocampus compared to the T2D group (*P*<0.0001) (Figure 6).

The results of One-way ANOVA revealed significant differences in TAU levels within the hippocampus among the groups (F _3,24 _=604, *P*<0.0001). Specifically, TAU levels in the hippocampus were significantly different between the CO and T2D groups (*P*<0.0001). Furthermore, the T2D+Ex group exhibited lower TAU levels in the hippocampus compared to the T2D group (*P*<0.0001) (Figure 6).

*MDA levels and GPx and SOD activity in the hippocampus*

The results of One-way ANOVA indicated significant differences in MDA levels within the hippocampus among the groups (F _3,24 _=16.73, *P*<0.0001). Specifically, MDA levels in the hippocampus differed significantly between the CO and T2D groups (*P*<0.0001). Additionally, the T2D+Ex group exhibited lower MDA levels in both serum and hippocampus compared to the T2D group (*P*<0.0004) (Figure 7).

The results of One-way ANOVA revealed significant differences in GPx (F _3,24 _=175.2, *P*<0.0001), SOD (F _3,24 _=89.3, *P*<0.001), levels within the hippocampus among the groups. Specifically, their levels were significantly different between the CO and T2D groups (*P*<0.01). Furthermore, the T2D+Ex group exhibited higher hippocampal levels of GPx, SOD (*P*<0.001), compared to the T2D group (Figure 7).

## Discussion

Our study aimed to investigate the impact of an 8-week HIIT regimen on lactate-induced mitophagy in the hippocampus of rats with type 2 diabetes (T2D). We demonstrated that HIIT resulted in improved mitophagy, consequently leading to a reduction in the accumulation of Aβ and tau proteins in the hippocampus of rats with T2D, in a lactate-dependent manner.

Mitophagy represents a selective autophagy process crucial for maintaining cellular homeostasis by specifically targeting and eliminating damaged or dysfunctional mitochondria (53). Lactate is capable of traversing the blood-brain barrier (BBB) via monocarboxylate transporter 2 (MCT2), reaching various brain regions, including the hippocampus. In this region, lactate has been shown to enhance neurogenesis and promote the improvement of mitochondrial quality through the activation of mitophagy (54, 55). Notably, lactate levels in the hippocampus were found to be higher in the T2D+EX group compared to the T2D group (56). Consistent with our study findings, several previous studies (57, 58) have demonstrated that during HIIT, lactate levels in the bloodstream can elevate substantially, reaching levels as high as 25 mmol·L−1. 

Measurement of transporters such as MCT2 is crucial because previous research has indicated that suppressing MCT2 can lead to a decrease in lactate levels in the hippocampus. This highlights the importance of understanding transporter dynamics in regulating lactate availability and its potential effects on brain function (59). A study demonstrated that HIIT results in elevated levels of MCT2 in the hippocampus. This increase in MCT2 expression was associated with improved brain function (12). Other studies (12, 59-61)have indicated that lactate itself can serve as a mediator for the effects of HIIT on the mitochondrial quality control system in the hippocampus. However, a study conducted by Zheng-Hong *et al*. (62) contradicts our findings and demonstrated that HIIT decreased the expression of mitophagy-related proteins in the hippocampus of middle-aged mice. The discrepancy between our findings and those of Zheng-Hong *et al*. may be attributed to the diabetic condition of the animals in our study. Mitophagy in diabetic conditions responds positively to exercise, potentially leading to more pronounced effects compared to healthy mice where mitophagy is not impaired (63).

Indeed, previous studies have shown that SIRT1 activation can lead to up-regulation of the FOXO1/3-PINK1-Parkin pathway, consequently enhancing the process of mitophagy (20, 21). Furthermore, it has been demonstrated that SIRT1 levels increase in response to the presence of lactate (15). In our study, we also measured the levels of SIRT1 and found that the results were consistent with previous research. HIIT increased SIRT1 levels in diabetic rats, possibly due to the positive response of SIRT1 to elevated lactate levels and an increased NAD+/NADH ratio. The elevation in SIRT1 can enhance the activity of the transcription factor FOXO3 through deacetylation (64). In our study, the levels of FOXO3 were found to be consistent with the levels of SIRT1 across the groups, which aligns with the findings reported by Askarian *et al*. (65). FOXO3 has been shown to directly enhance the expression of antioxidant enzymes (46). Consistent with this, our study revealed increased levels of antioxidant enzymes such as SOD and GPx, along with a decrease in oxidative stress markers such as MDA, in the T2D+EX group compared to the T2D group (65). FOXO3 can also play a role in inducing mitophagy by binding to the regulatory sites of the proteins PINK1 and Parkin (20). Indeed, PINK1 functions as a kinase protein, while Parkin acts as a ubiquitin ligase E3. Together, they regulate the selective removal of dysfunctional or surplus mitochondria through mitophagy. PINK1 governs the translocation of Parkin to damaged mitochondria, thereby initiating the process of mitophagy and facilitating the elimination of impaired mitochondria (66). It has been demonstrated that Parkin plays an essential role in initiating exercise-induced mitophagy (67). Chen et al. demonstrated that endurance exercise significantly reduces exercise-induced mitophagy, possibly attributable to improved mitochondrial quality (67). In our current study, we observed an increase in factors associated with mitophagy in the HIIT exercise groups, which may be attributed to a higher accumulation of ROS in these groups. Previous research has indicated that ROS accumulation can stimulate mitophagy. It is plausible that exercise-induced mitophagy is triggered by inflammation and ROS accumulation, while regulated mitophagy can help eliminate ROS and mitigate inflammation. Consequently, mitochondrial damage may be attenuated (68). Interestingly, in another study, an increase in the capacity for mitophagy was observed after an 8-week continuous moderate training regimen (69). During mitophagy, cytosolic LC3 is indeed conjugated with phosphatidylethanolamine, resulting in the formation of LC3-II, which is subsequently recruited to autophagosome membranes. The increase in LC3 levels indirectly indicates the activation of mitophagy, as observed in our study.

Recent studies have delved into the role of impaired mitochondrial function and bioenergetic deficits in the accumulation of amyloid-beta (Aβ) and tau proteins in the brains of patients with cognitive impairment. These findings suggest that mitochondrial dysfunction represents an early event in the pathogenesis of cognitive impairment (70). Indeed, when mitophagy is impaired, dysfunctional mitochondria accumulate, resulting in heightened oxidative stress. Moreover, the potential of mitochondrial membranes decreases, which can lead to mitochondrial damage and further exacerbate oxidative damage (7). Our results demonstrated that high-intensity interval training (HIIT) could decrease oxidative stress and enhance antioxidant defense mechanisms in rats with type 2 diabetes (T2D). These findings align with previous research in this area (12, 26, 28).

Our study clearly demonstrated that the accumulation of amyloid-beta (Aβ) and tau proteins in the hippocampus was significantly higher in the T2D group compared to other groups, and high-intensity interval training (HIIT) significantly reduced the levels of Aβ and tau. This reduction could be attributed to the enhancement of the mitophagy process. Consistent with our findings, several other studies have also shown that HIIT can reduce the accumulation of Aβ and tau by improving the mitophagy process (71, 72). A study demonstrated that high-intensity interval training (HIIT) could decrease mitophagy, which contrasts with our study findings. The disparity in results may stem from differences in the experimental model, as their study was conducted on mice, whereas ours was conducted on rats. Furthermore, variations in the duration and frequency of HIIT sessions could also contribute to differences in results. It is possible that longer exercise periods may lead to greater adaptations, including alterations in monocarboxylate transporter 2 (MCT2) expression (62). Although lactate levels are elevated in the serum of diabetic individuals, impairment in mitophagy has also been observed in these individuals. This discrepancy could be attributed to a deficiency in lactate transporters, such as monocarboxylate transporters (MCTs), in diabetes. Reduced levels of lactate transporters may limit the entry of lactate into various tissues, including the brain and hippocampus, which are crucial for mitophagy pathways and lactate signaling (14, 15).

**Figure 1:**
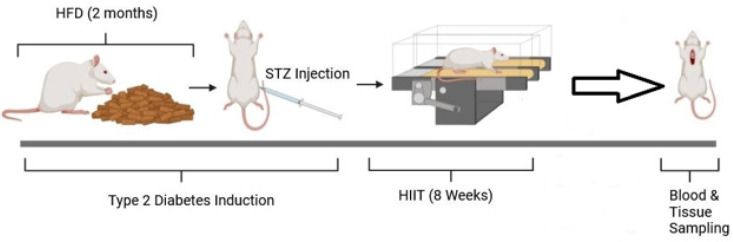
Study timeline

**Figure 2:**
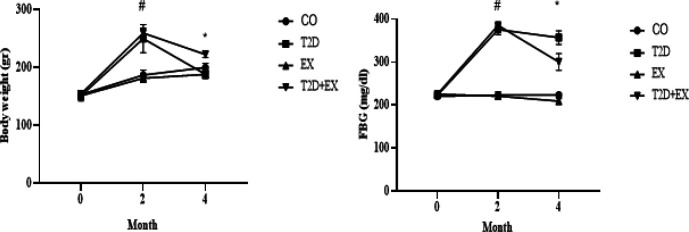
Body weight and Fasting blood glucose (mean±SD) before starting the intervention (month 0), after diabetes induction (2 months of high-fat diet and STZ injection) (month 2), and 48 hr after the last training session (month 4) in all groups (n=7)

FBG: Fasting blood glucose Con: control, T2D: Type 2 diabetic (STZ injected), Ex: exercise only, and T2D+Ex: Type 2 diabetic+ exercise. # significant difference between T2D and T2D+Ex with other groups, * significant difference between T2D and T2D+Ex

**Figure 3:**
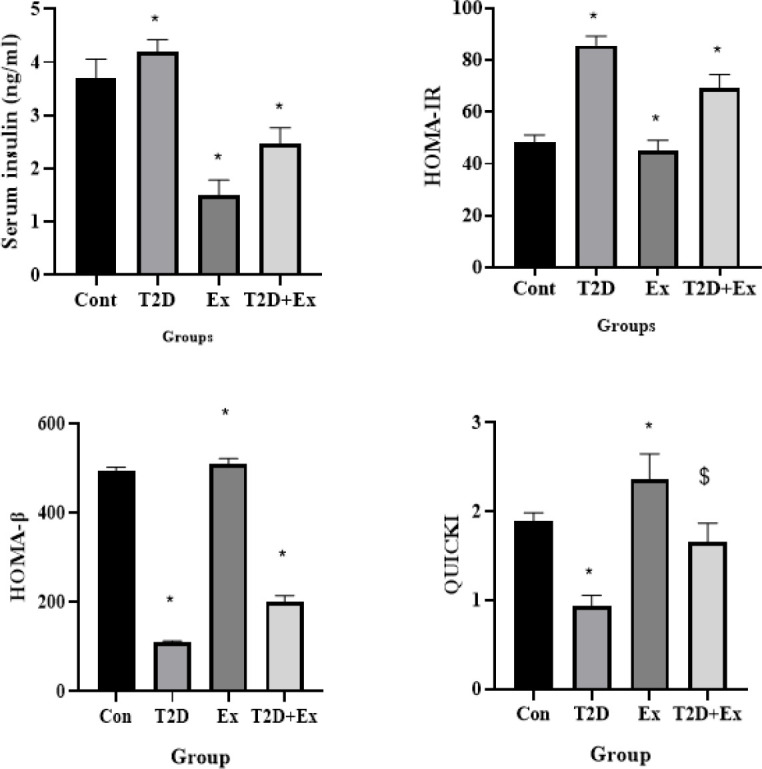
Insulin, HOMA-IR, HOMAβ, and QUICKI

Con: control, T2D: Type 2 diabetic, Ex: exercise, and T2D+Ex: Type 2 diabetic+ exercise. * Significant difference with other groups. $ Significant difference with the T2D group.

**Figure 4:**
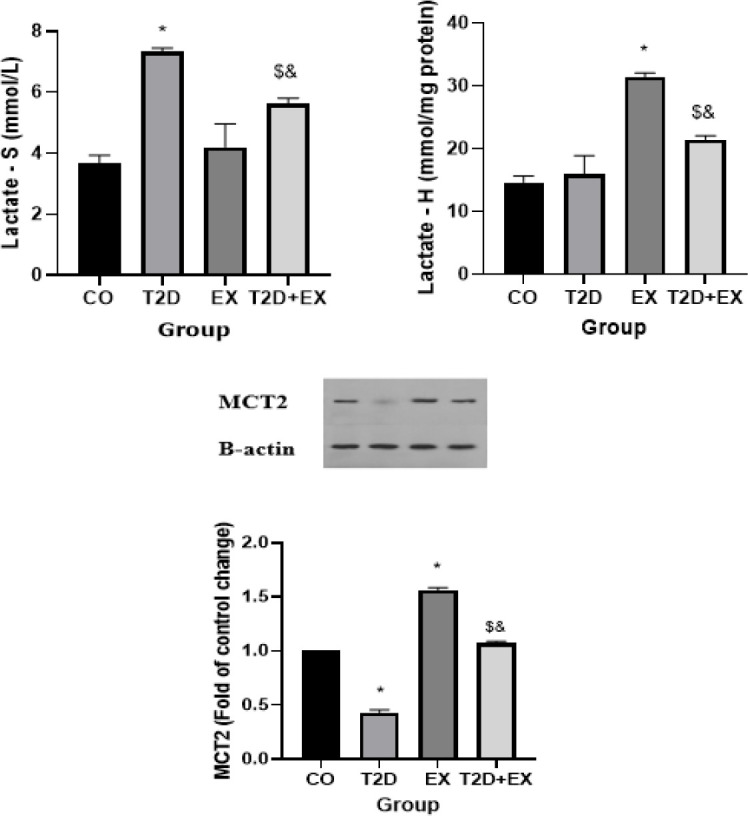
Lactate levels in serum (lactate-Sand hippocampus (lactate-H) and MCT2 levels in the hippocampus (n = 7 in each group)

CO: Control, T2D: Type 2 diabetic, EX: Exercise and T2D + EX: Type 2 diabetic +Exercise

* Significant difference with all groups (*P*<0.001), & Significant difference with T2D group (*P*<0.001), $ Significant difference with Ex group (*P*<0.001).

**Figure 5:**
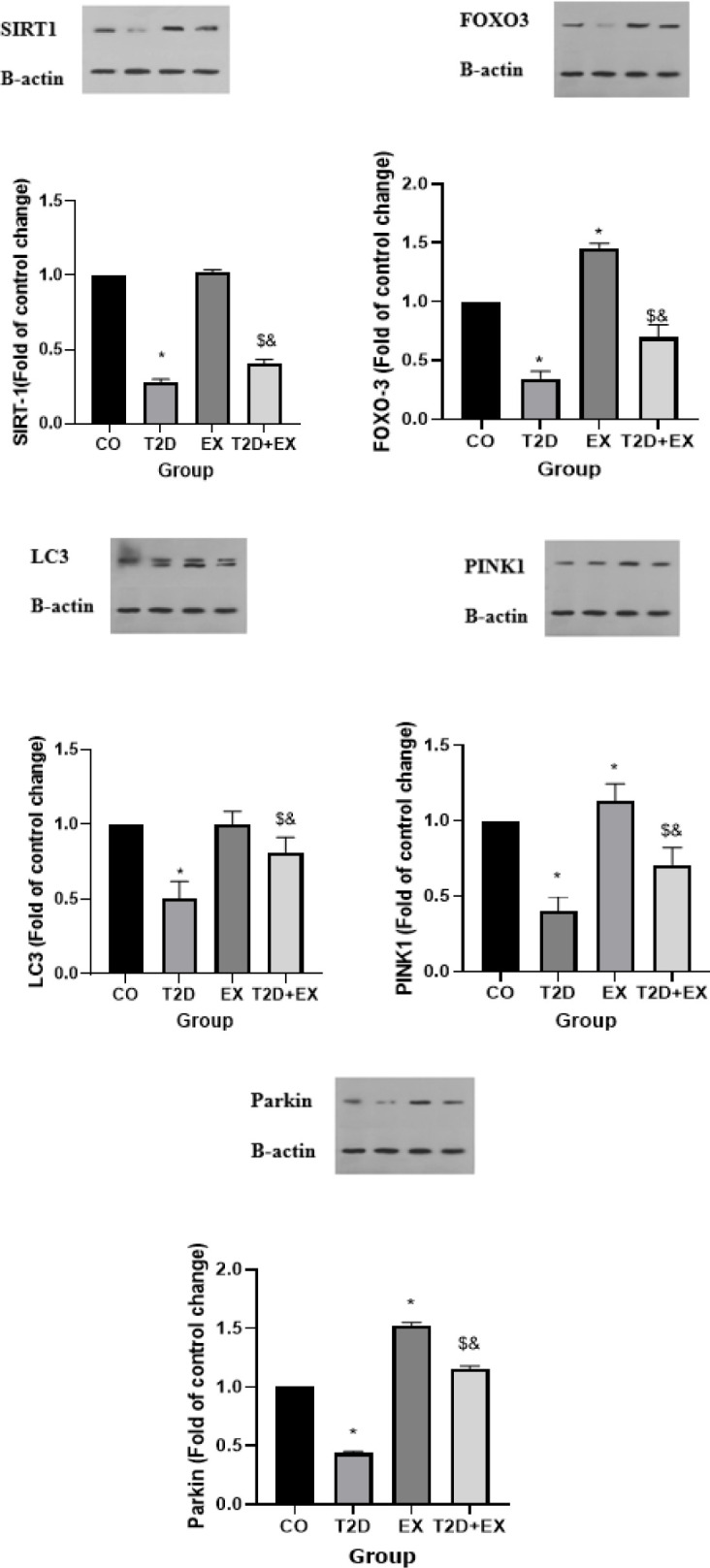
SIRT-1, FOXO-3, PINK1, Parkin, and LC3 levels in the hippocampus (n= 7 in each group)

CO: Control, T2D: Type 2 diabetic, EX: Exercise and T2D + EX: Type 2 diabetic +Exercise

* Significant difference with all groups (*P*<0.001), & Significant difference with T2D group (*P*<0.001), $ Significant difference with Ex group (*P*<0.001).

**Figure 6:**
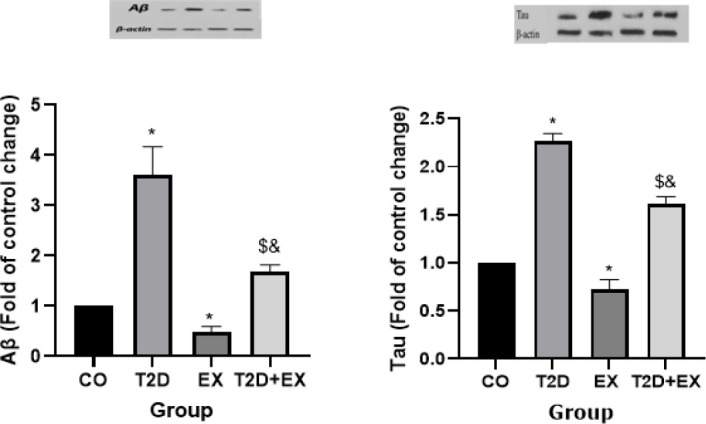
Aβ and Tau levels in the hippocampus (n= 7 in each group)

CO: Control, T2D: Type 2 diabetic, EX: Exercise and T2D + EX: Type 2 diabetic +Exercise

* Significant difference with all groups (*P*<0.001), & Significant difference with T2D group (*P*<0.001), $ Significant difference with Ex group (*P*<0.001).

**Figure 7:**
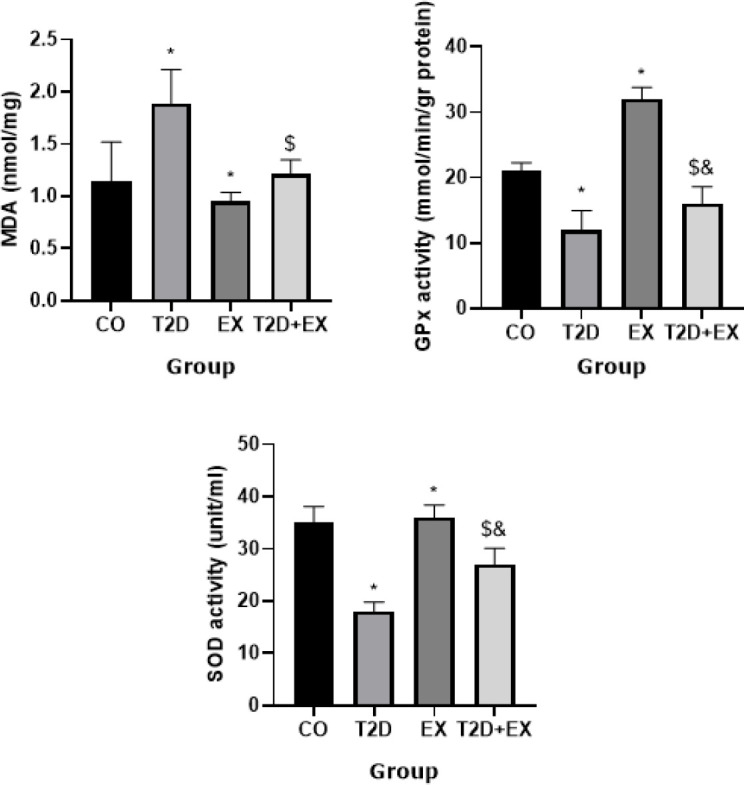
MDA levels, and GPx and SOD activity in the hippocampus (n= 7 in each group)

CO: Control, T2D: Type 2 diabetic, EX: Exercise and T2D + EX: Type 2 diabetic +Exercise

* Significant difference with all groups (*P*<0.001), & Significant difference with T2D group (*P*<0.001), $ Significant difference with Ex group (*P*<0.001).

## Conclusion

High-intensity interval training (HIIT) represents a promising non-pharmacological intervention that may enhance lactate signaling in the hippocampus of diabetic rats. This enhancement of lactate signaling could lead to improvements in mitochondrial quality through the lactate-SIRT1-FOXO3-PINK1/Parkin pathway. Consequently, this pathway may contribute to the reduction of Aβ and tau accumulation in the hippocampus.

## Data Availability

Data that support the findings of this study are available at Shahid Rajaei Teacher Training University.

## Study Limitations and Suggestion for Future Studies

Since multiple pathways, including the Parkin-independent pathway, contribute to the process of mitophagy and may have implications for preventing cognitive impairment, it is advisable for future studies to explore these pathways. Investigating a comprehensive range of mitophagy pathways could provide valuable insights into potential therapeutic strategies for cognitive decline.
